# A cost savings analysis of asynchronous teledermatology compared to face-to-face dermatology in Catalonia

**DOI:** 10.1186/s12913-018-3464-4

**Published:** 2018-08-22

**Authors:** Josep Vidal-Alaball, Josep Lluís Garcia Domingo, Francesc Garcia Cuyàs, Jacobo Mendioroz Peña, Gemma Flores Mateo, Joan Deniel Rosanas, Glòria Sauch Valmaña

**Affiliations:** 1 Health Promotion in Rural Areas Research Group (PRoSaARu), Gerència Territorial de la Catalunya Central, Catalan Health Institute, Carrer Pica d’Estats, 36, 08272 Sant Fruitós de Bages, Barcelona Spain; 2 Unitat de Suport a la Recerca de la Catalunya Central, Institut Universitari d’Investigació en Atenció Primària Jordi Gol (IDIAP Jordi Gol), Barcelona, Spain; 3 Department of Economics and Business, University of Vic- Central University of Catalonia, Vic, Barcelona Spain; 4 General Surgery Department, Autonomous University of Barcelona, Barcelona, Spain; 5 Chair in ICT and Healthcare, University of Vic-Central University of Catalonia, Vic, Barcelona Spain; 6 Xarxa Sanitària i Social de Santa Tecla, Tarragona, Spain

**Keywords:** Teledermatology, Telemedicine, Telehealth, Cost savings analysis, Primary care

## Abstract

**Background:**

A teledermatology pilot scheme was first conducted in the town of Manresa (Barcelona) in the summer of 2010. The clinical success of the scheme prompted its expansion to the whole county of Bages in 2011 and to the adjacent county of Berguedà in 2012.

In the teledermatology service, primary care physicians take a photograph of the lesion and attach it to the electronic medical records of the patient together with a brief clinical account. In the referral hospital, the consultant dermatologists access the electronic medical records, review the images and suggest a treatment or action plan. Next, the primary care physicians review these recommendations and call the patient to report the results. This whole process is usually completed in less than 5 working days.

**Methods:**

A cost saving analysis comparing teledermatology with dermatology face-to-face visits was performed in the county of Bages measuring the cost difference attributable to visits saved.

**Results:**

The estimated added costs of the teledermatology service during 2016 amounted to 61,870 €. For the same period, the estimated costs of traditional outpatient dermatology services were of 113,034 €. This represents savings of 51,164 € per year. After subtraction of societal costs, the savings equal 10,350 € per year.

**Conclusions:**

Using a teledermatology service instead of face-to-face dermatology consultations could save 51,164 € per year (11.4 € per patient visited) in the county of Bages. Societal savings are the most significant.

## Background

Telemedicine can be defined as “*the use of telecommunications technology to provide medical information and services*” [1] or as “*medicine practiced at a distance*” [2]. There are three main types of telemedicine: (1) real-time or synchronous telemedicine; (2) not real-time or asynchronous telemedicine; and (3) remote patient monitoring [3, 4].

In the last 25 years, telemedicine services have been implemented in many countries. In 2010, a World Health Organization survey found that 38% countries provided some kind of telemedicine and 30% countries had agencies that included telemedicine services [5]. Globally, teleradiology is the most common telemedicine service, followed by telepathology and teledermatology [5].

In Manresa (Barcelona), telemedicine was first introduced as a teledermatology pilot scheme in the summer of 2010 to solve the rising dermatology waiting list aggravated by generalized healthcare cuts. Early evidence of positive clinical impact and acceptance by professionals prompted the expansion of teledermatology services to all the county of Bages in 2011 and to the adjacent county of Berguedà in 2012. In 2014, a study on the impact of teledermatology on decreasing dermatology waiting lists in the Bages region during the 2009–2012 period was published. The results showed a reduction in dermatology waiting times from a mean of 30 days (95% CI: 29–32) before the implementation of teledermatology to a mean of 16 days (95% CI: 15–17) after its implementation [6]. The county of Bages is located in the centre of Catalonia, with a population of 184,403 inhabitants (91,260 men and 93,143 women) and a surface area of 1299.1 km^2^. It has a lower population density (141.9 inhabitants/Km^2^) than the average of Catalonia (234.2 inhabitants/Km^2^) [7].

When primary care physicians decide to use the teledermatology service, they take a photograph of the lesion and attach it to the electronic medical records of the patient together with a brief clinical account. The use of electronic medical records guarantees the security of the images, since it avoids potentially insecure electronic storage systems and email. The dermatologists in the hospital access the electronic medical records, review the images and suggest a treatment or action plan. Next, the primary care physicians review these recommendations and phone the patient to explain the results of the consultation. The whole process takes usually less than 5 working days. The dermatologist might occasionally ask the primary care professional to refer the patient for a face-to-face visit. Except for urgent cases, the teledermatology service can be used for all dermatological conditions and the follow up is mostly performed by general practitioners.

The main objective of this study was to assess the economic impact of asynchronous telemedicine services in the Catalan central region comparing the cost of teledermatology with the cost of traditional outpatient consultations to determine whether to expand the service to other regions of Catalonia. Cost savings are estimated per patient visited and extrapolated to the whole Catalan territory.

## Methods

A cost saving analysis comparing teledermatology with traditional dermatology consultations in the county of Bages was performed measuring direct and indirect costs and the cost of the visits saved. This analysis was selected because there is evidence that the effectiveness of traditional outpatient consultations and asynchronous teledermatology is the same [8, 9].

The population of reference included all patients allocated to the 14 Primary Health Care Teams in the county of Bages.

### Data collection

The Catalan Institute of Health provided anonymized quantitative data regarding patients and number of visits to traditional outpatient dermatology services and teledermatology during 2016. Secondary data were obtained from the literature and expert opinion.

### Number of visits

Data regarding number of dermatology visits, teledermatology visits and number of traditional outpatient visits after an initial teledermatology consultation were analysed.

The number of traditional outpatient consultations saved by the teledermatology service was calculated by subtracting the number of outpatient visits requested after an initial teledermatology consultation from the total number of teledermatology consultations.

### Identification of costs

Costs directly attributable to teledermatology and to the traditional dermatology service included cameras, hardware and staff. Costs not directly attributable to teledermatology and to the traditional dermatology service included building maintenance, Information technology (IT) services, gas, electricity, telephone-internet connections and medical insurance. Costs incurred by patients and society such as lost productive time, lost salaries, leisure time lost, time spent travelling to visits and petrol were also considered.

### Equipment costs

The market cost of an iPad Air with Wi-Fi and Cellular with 32GB was used [10].

### Staff costs

To calculate the hourly rate of a primary care physician, we used the average (37,982 € per year in 2015) of the highest and lowest salaries paid by the Catalan Institute of Health, the main provider of primary care services in Catalonia. Taking into consideration that a primary care physician works 1642 h per year, the estimated hourly rate of this professional was 23.1 € [11]. To calculate the hourly rate of a dermatologist, the average (34,574.38€ per year in 2015) of the highest and lowest salaries agreed in the Collective Work Agreement used by the majority of hospitals in Catalonia was used. Taking into consideration that a dermatologist works 1688 h per year, the hourly rate of this professional was estimated at 20.5 € [12].

### Productivity loss

The shortest time indicated in Google maps to travel by car from the village or town where the patient lives to the hospital located in Manresa (Hospital Sant Joan de Déu, C/ Dr. Joan Soler, s/n, 08243 Manresa) was used.

The time spent travelling to the hospital and the average hourly cost of labour was used to calculate the lost productive time. The average hourly wage in the last quarter of 2015 was used (21.2 €) [13]. To account for the time spent in consultation with the dermatologist (15 min on average for first visits in the local hospital according to the dermatology consultants) [14] and the waiting time before entering this consultation, an average of 25 min was added.

We considered that most patients live in the town of the primary care centre where the photograph was taken, and therefore no travelling time was added. An average of 20 min was added to account for the time spent in consultation with the general practitioner (10 min plus waiting time).

### Travel cost

To calculate the cost of petrol to drive to the hospital, the average price of mileage paid by companies in Spain in 2015 was used. Since this price ranges from 0.07€/km to a maximum of 0.75€/km, the average was calculated at 0.25€/km [15]. The mileage by car from the patient’s address to the Hospital in Manresa was calculated using Google maps. Because in the county of Bages many towns and villages do not have regular public transport service, it was assumed that private transport was used on all occasions.

### Costs excluded

Although friends and relatives of the patient are often affected by the patient’s condition, it is still unclear how to account for this specific item [16]. In this study, the costs to patient companions were excluded, since information on whether the patients went to the consultations on their own or accompanied was unavailable.

The costs of lost leisure time were also excluded because we could not differentiate whether working hours or leisure hours had been lost, and it was assumed that consultations in hospital and in primary care took place during working hours.

Training costs were excluded because no extra training was provided for family care physicians and dermatologists. Structural costs, technical costs and medical insurance costs were excluded since they were not considered significant. Table 1 shows excluded costs.

**Table 1:** Costs excluded and reason for exclusion

Costs	Reason for exclusion
Technical costs	No significant added costs^a^
Building maintenance	No significant added costs^a^
Electricity, heating	No significant added costs^a^
Medical insurance	No significant added costs^a^
Telephone calls	No significant added costs
Training costs	No significant added costs
Time of patient companions	Very difficult to quantify through secondary data

^a^Teledermatology represents 1.4% of the total activity of the hospital [17]

## Results

### Number of visits saved

During 2016, 5606 patients were referred to the teledermatology service, of which 1104 patients were further referred to traditional outpatient consultation (within the next 3 months). Consequently, the teledermatology service saved a total of 4502 face-to-face visits.

### Equipment costs

It was assumed that if each of the 14 primary care practices in the Bages area acquired an iPad Air (Wi-Fi + Cellular 32GB) at a price of 559 € each, the initial cost of this equipment would be of 7826 € (559 € × 14 = 7826 €). The equipment is expected to last approximately 5 years. Taking into account this obsolescence, the annual cost of this equipment was calculated at **1565 €**.

### Primary care physician costs

During 2016, 4502 visits were potentially saved by the use of the teledermatology service. Since a teledermatology primary care appointment needs an initial 10 min consultation with the primary care physician, a total of 45,020 min (750.3 h) were spent in teledermatology-related appointments. The estimated hourly rate of this professional was calculated at 23.1 €, therefore the cost of the total time spent in teledermatology-related consultations during 2016 was **17,333 €**. After the initial consultation and having received the dermatologist’s recommendations, the primary care physician contacted the patient by phone to explain the further management of the condition. This phone call was estimated to last an average of 2 min and thus a total of 9004 min (150.1 h) were spent in teledermatology-related telephone calls in 2016. Since the hourly rate of the GP was 23.1€, the cost was calculated at **3467 €** per year. The total costs of the time spent by primary care physicians in teledermatology-related appointments was **20,799 €** during 2016.

### Dermatologist costs

During 2016, 4502 visits were potentially saved by the teledermatology service. Assuming that a traditional face-to-face dermatology appointment requires a 15 min consultation, a total of 67,530 min (1125.5 h) would have been spent in traditional dermatology appointments during 2016 if the teledermatology service had not been in place. With the estimated hourly rate of dermatologists calculated at 20.5 €, the cost of this time would have been **23,073 €** per year.

Considering that a teledermatology appointment needs a 5-min consultation by a dermatologist, a total of 22,510 min (375.2 h) were spent in teledermatology-related appointments. With the estimated hourly rate of this professional at 20.5 €, the cost of this time was calculated at **7691 €** per year.

Table 2 shows the cost of primary care physicians and dermatologists.

**Table 2:** Cost of primary care physicians and dermatologists

Service		Primary care		Dermatology	
		Initial consultations	Telephone calls	Face-to-face	Teledermatology
N. Visits	min	45,020	9004	67,530	22,510
4502	hours	750,3	150,1	1125,5	375,2
	Cost	17,333	3467	23,073	7691

### Cost of time lost

The time spent travelling to the hospital to visit the dermatologist was calculated for each of the primary care centres, adding 25 min for the visit plus the waiting time. During 2016, a total of 2840 h would have been spent if all patients had attended a hospital visit instead of using teledermatology. Since the average hourly wage was 21.2 € [13], the total cost for 2016 would be **60,208 €**.

The time spent travelling to the primary care centre to take a photograph, adding 20 min for the visit and the waiting time, was estimated at 1500.7 h for 2016. Considering that the average hourly cost of labour is 21.2 €, the total cost was **31,815 €**. Table 3 shows cost of time lost by primary care centre.

**Table 3:** Cost of time lost by primary care centre

	TeleDM Visits	Referrals	Saved visits	Time to hospital	Time spent in hospital		Time spent in primary care	
				(mins)	(25 mins)	Hours	(20 mins)	Hours
CAP MANRESA 2	1067	227	840	9	28,560	476	16,800	280,0
CAP SAGRADA FAMILIA	828	178	650	3	18,200	303,3	13,000	216,7
CAP SALLENT	376	53	323	15	12,920	215,3	6460	107,7
CAP SANT FRUITÓS	294	64	230	7	7360	122,7	4600	76,7
CAP SANT JOAN DE VILATORRADA	494	105	389	11	14,004	233,4	7780	129,7
CAP SANT VICENÇ DE CASTELLET	345	69	276	12	10,212	170,2	5520	92,0
CAP SANTPEDOR	286	59	227	11	8172	136,2	4540	75,7
Other	1916	349	1567			1183		522,7
Total	5606	1104	4502		Total time	2840		1500,7
					Total €	60,208		31,815

### Cost of petrol

Table 4 shows that patients would have travelled a total of 49,684 km if they had attended traditional outpatient consultations instead of using teledermatology. This amounts to **12,421 €** in petrol.

**Table 4:** Cost of petrol by primary care centre

Primary care centre	Saved visits	Km to Hospital	Saved Km	Cost Km saved
CAP MANRESA 2	840	2,8	2352	588
CAP SAGRADA FAMILIA	650	1,2	780	195
CAP SALLENT	323	15	4845	1211
CAP SANT FRUITÓS	230	6	1380	345
CAP SANT JOAN DE VILATORRADA	389	9	3501	875
CAP SANT VICENÇ DE CASTELLET	276	9,2	2539	635
CAP SANTPEDOR	227	9,2	2088	522
Other	1567		32,199	8050
Total	4502		49,684	12,421

### Total costs

Table 5 shows that the estimated added costs of the teledermatology service during 2016 were of **61,870 €.** For the same period, the estimated costs of the traditional outpatient services if all patients had been referred to face-to-face visits would have been of **113,034€.** This represents cost savings of **51,164 €** during 2016. Since in 2016 a total of 4502 patients used these services, the savings amount to 11.4 € per patient visited.

**Table 5:** Annual costs of teledermatology compared with traditional outpatient dermatology consultations

Cost per year in €	Teledermatology	Dermatology	Difference
Equipment	1565	0	1565
Primary care staff	20,799	17,332	3467
Hospital staff	7691	23,073	−15,382
Subtotal	30,055	40,405	−10,350
Society			
Time	31,815	60,208	−28,393
Petrol		12,421	−12,421
Total	61,870	113,034	−51,164

In the analysis, societal costs emerge as the variable with the biggest impact on our calculations; savings due to teledermatology amounted to **40,814 €** per annum. The main savings derived from time saved by not travelling to the hospital. When removing societal costs, teledermatology savings amounted to **10,350 €** in 2016.

Staff costs were also significant, particularly in the hospital, since the use of teledermatology saved considerable time to dermatology consultants. With traditional outpatient consultations, staff costs in hospital amounted to 23,073 € per year, whereas with teledermatology this amount was reduced to 7691 € per year (annual savings of 15,382 €). In primary care, staff costs increased slightly with teledermatology because primary care physicians were required to phone patients to explain the results. With conventional dermatology, the staff costs in primary care were 17,332 € per year, whereas with teledermatology costs increased to 20,799 € per year (annual increase of 3467 €).

## Discussion

This cost savings study has compared the marginal cost of the resources associated with the use of a teledermatology programme in the Catalan Central Region with the cost of face-to-face dermatology consultations in order to elucidate whether teledermatology generates savings.

The analysis suggests that the teledermatology programme implemented in the Catalan Central Region could generate important cost savings (up to 51,164 € per year) when compared with the traditional outpatient consultation model.

These results are consistent with other studies. Armstrong et al. published in 2007 an economic evaluation comparing the hourly costs of a teledermatology service with a face-to-face dermatology clinic in the United States. They concluded that the hourly cost of operating the teledermatology practice was lower than that of the conventional clinic [18].

A number of limitations need to be taken into consideration when evaluating the results of the study. Firstly, we have compared the cost of teledermatology versus traditional outpatient consultation using our day-to-day clinical experience, data obtained from experts and a review of the literature. Secondly, it is unclear why some patients are referred for face-to-face dermatology visits after an initial teledermatology consultation and whether they attend them.

The costs excluded and included are amongst the most controversial aspects of economic studies. The current study excluded general costs of regular clinical practice. It also excluded costs considered similar between teledermatology and traditional dermatology practice and costs difficult to quantify through secondary data such as the costs to patient companions, costs of leisure time lost and costs of carbon emissions. These decisions are justified in the methods section.

Despite evidence that the clinical effectiveness of teledermatology is comparable with face-to-face consultations, some authors have recently raised concerns about the methodologies that determine the effectiveness of telemedicine [19].

This health economic evaluation assumed that the resources saved would be efficiently allocated to provide other dermatology services. If that were not the case, savings would be considered negligible.

In 2010 Eminović et al. conducted a cost minimisation analysis in store-and-forward teledermatology. The authors calculated that teledermatology was 32.5 € (95% CI, − 29.0 to 74.7) more expensive than conventional dermatology visits. They concluded that teledermatology could only generate savings if the distance to a dermatologist in hospital was larger (≥75 km) or when more consultations (≥37%) could be prevented with the use of teledermatology. In consequence, teledermatology should only be applied in those cases with a reasonable probability that a face-to-face consultation could be prevented [20]**.** The study included similar costs to ours and additional training for primary care physicians and dermatologists and costs of diagnostics and treatment. They also included the societal costs of travel of the patient and a patient companion based on the estimate that about 20% of patients (children and elderly) visit a health professional accompanied.

The setting of this study is the county of Bages. However, we believe that the data can be extrapolated to the other regions of Catalonia. We calculated that the savings for the whole population of Catalonia (7,519,000 inhabitants) would amount to 2,085,061 €. Caution should be exercised when extrapolating these results to other countries, since different fees apply to different health systems.

## Conclusions

The results of this study show that using teledermatology instead of face-to-face dermatology consultations can save 51,164 € per year (11.4 € per patient visited) in the county of Bages. Most savings were societal (40,814 € per year). When removing societal costs, the savings amounted to 10,350 € per year.

## Abbreviations

IT: Information technology
TeleDM (table): Teledermatology
